# On the Analogy between Natural Small-Pox, Inoculation, and Vaccination

**Published:** 1815-11

**Authors:** Richard Walker

**Affiliations:** of Oxford.


*For the London Medical and Physical Journal.*

*On the Analogy between Natural Small-pox, Inoculation, and Vaccination;* by RICHARD WALKER, Esq. of Oxford.

(*Continued from p.* 301.)

THE only essential difference, according to ordinary observation, between inoculation and vaccination, is, that the former produces pustular eruptions exactly analogous to natural small-pox; the latter no eruptions, ordinarily. Instances, however, sometimes occur in inoculation, where the progress of the inoculated part has been perfect, but no variolous eruption has ensued: such cases, however, are not considered so satisfactory, and, it is known, have been succeeded by natural small-pox, at some distant period, notwithstanding the confident promises of perfect security given by some practitioners to their patients under such circumstances.

The nature of the succeeding affection, both in inoculation and vaccination, may be ordinarily anticipated, or foretold, by the nature of the pustule in the part where the matter is inserted, at its commencement, or early in its progress, viz. the mild or severe kind of pustule before described being succeeded by a more gentle or more violent affection. Moreover, as the affection from vaccination is at all times harmless, respecting the welfare of the patient, the more highly inflamed pustule, in this instance, is more desirable, as it is thought satisfactory.

N.B.—It sometimes happens, as is well known, that in inoculation for small-pox, a very full eruption occurs, which turns out to be a mere rash, and which quickly disappears. It is of use to be able to distinguish these from the genuine variolous eruptions with which they are ordinarily intermixed. These eruptions do not exhibit the minute vesicle which soon appears in the variolous eruptions.

It is a well-established fact, and which I have repeatedly experienced myself, that matter taken from the confluent kind of small-pox may communicate to others, by inoculation, the distinct small-pox; and *vice versa,* matter from the distinct kind, the confluent. The same circumstance occurs respecting small-pox taken in the natural way; and, likewise, in transferring the more virulent kind of matter in vaccination, the milder kind may be produced, and *vice versa;* tending to prove, that the matter is essentially the same in each instance, and that the difference depends upon difference of habit. Inoculation, however, possesses a specific effect in producing a milder kind of small pox, than the casual mode of taking the disease: this is, probably, owing to its being received in a more partial or less general manner. In consequence of this specific effect in inoculation, the confluent kind of small-pox in it is a very rare occurrence.

The chances of taking small-pox after having passed through vaccination will depend upon the following contingencies, viz. the greater or less degree of idiosyncrasy, or susceptibility in the habit for receiving small-pox (certain persons being capable even of taking the natural small-pox a second time*); the length of time passed since; and the degree of infection to which the person is exposed.

There is evidently, as I have ascertained, a regular gradation of variolous affection, or small-pox, from the highest or most virulent kind of confluent small-pox, down to the mildest degree of the distinct kind.

This circumstance was most satisfactorily exemplified in the several persons of the family before alluded to. The

---

* There is no reason to doubt of this fact; but it is not improbable that in some instances chicken-pox may have been mistaken for small-pox. The chicken-pox is sometimes so like the mild kind of small-pox, that even practitioners themselves are at a loss to discriminate. From this circumstance, I apprehend, some errors have arisen respecting small-pox after vaccination. The pustules in chicken-pox, it must be admitted, in some instances, resemble pretty closely those of small-pox; but in addition to the ordinary means of discriminating them, viz. the slightness of the indisposition, the shorter duration of the pustules, in chicken-pox, and their early scabbing, I would observe that they never exhibit that perfectly well-defined hemispherical figure, which constantly occurs in the pustules of distinct small-pox; nor do they at any time acquire, like these, the truly purulent appearance, the fluid exhibiting more the appearance of a yellowish serum.

It sometimes happens, indeed, that variolous pustules, unquestionably genuine, being, in some instances, the effect of inoculation from a person loaded with small-pox, as was the case in the boy who had been previously vaccinated, never proceed to maturation; but even here there is that well-defined tubercular appearance which clearly distinguishes them from chicken-pox.

two two children who had been neither inoculated nor vaccinated, had the confluent small-pox to so violent a degree that one died, and the other just struggled through it. Of the three that had been vaccinated, two took the small-pox naturally, which was of the distinct kind, and the other from inoculation, who sickened in the ordinary way, and had a variolous eruption of about a dozen in number, so mild, that neither of them suppurated; and the mother, who had had the natural small pox many years ago, had several eruptions, evidently variolous, some of which suppurated, and would doubtless have communicated the small-pox to another person by inoculation.

It is a fact well ascertained, that, if a person who has had the natural small-pox be inoculated, the inserted virus may produce a variolous pustule, the matter of which will produce small-pox in another person.

It is worthy of notice, that the mother of these children declares that herself, when a child, with the rest of the family, at that time, had the small-pox naturally, and in a very severe manner. It is well known that an idiosyncrasy of this nature frequently pervades a whole family.

The boy that had the small-pox from inoculation in so mild a manner now, after having passed through vaccination four or five years ago, has a scar left from the former affection, sufficient to receive a silver penny. This, in conjunction with the other circumstances mentioned, tends to shew, or prove, that consequent small-pox, should it occur, is ameliorated or diminished in proportion to the degree of idiosyncrasy in the habit for receiving it, and in proportion to the perfection of the former affection, or, in other words, according to the ratio of abstraction of the essence, or innate principle (if I may be allowed to call it so), originally existing in the habit. Moreover, it is remarkable, that this boy had escaped the natural affection considerably beyond the time it is probable he could have taken it from the rest; and was inoculated now merely for putting to a further test the vaccination, which had withstood this exposure to small-pox. This boy lived in the same house, but did not sleep with the infected children, as the others did; and had, as appears, but a very small portion of the variolous essence, or latent principle of the disease, remaining to be evolved or thrown off.

An instance has likewise occurred here, in which a child who had passed through vaccination in a perfect manner, five or six years ago, was at this time, by way of test, inoculated with variolous fluid, when the inoculated part rose and passed through the usual progress; and the child was evidently evidently indisposed, though but slightly, about the usual time, viz. the seventh day, but in this instance it went no farther; there was no eruption.

I have likewise met with an instance where the mother, during her attendance on a child loaded with confluent small-pox, not only had variolous eruptions, but a sensible indisposition previously; notwithstanding having passed through small-pox in a perfect manner before. Strange as it may appear that three children, in one family, who had passed through vaccination four or five years ago, (these being vaccinated at different periods of time, and consequently with vaccine virus taken from different subjects) should be incident to small-pox, being exposed to the most powerful means of infection; all wonder must cease when Mr. Hugo mentions an instance where four children out of five in the same family, who unquestionably had passed through vaccination in the most perfect manner, were attacked subsequently by genuine small-pox; and, upon the whole, he enumerates twenty-five persons who had been duly vaccinated by different practitioners, and ordinarily with the precaution of two punctures, who, some time afterwards, being exposed to a more than ordinary degree of virulent small-pox, became susceptible of its infection, but for the most part of an unusually mild nature: and, moreover, even two persons who had several years before passed through inoculated small-pox in a satisfactory way.*

All these circumstances, taken collectively, abundantly confirm the fact respecting a regular chain of gradation of natural small-pox, from the most virulent or confluent kind, down to the very mildest degree of it; and that this difference depends, not upon the specific nature of the variolous fluid itself, but upon other circumstances, as idiosyncrasy of habit, the different mode by which it is received, and previous preparation of the subject by medicine and regimen.† And likewise accounts for the possibility of a recurrence of natural small-pox in the same person, as well as for the circumstance, though an extremely rare occurrence, of natural

* See Mr. Hugo's paper "On Small-pox succeeding Vaccination at Crediton," Med. and Phys. Journal, No. 190.

† The state of the air has likewise much influence respecting the severity or mildness of the affection, particularly when epidemic; extreme close hot weather being most strongly conducive to producing the more virulent, confluent, or malignant kind, which effect is increased much among the poorer class of people, in consequence of their being crowded, and sleeping together, in close or small apartments.

smallsmall-pox succeeding inoculation; and the less rare, though very far indeed from an ordinary occurrence, or one to be ordinarily apprehended, an attack of small-pox after perfect vaccination.

In a former paper, I have ventured an opinion that the innate principle of small-pox exists originally in the habit, and that a person may be still susceptible of taking small-pox so long as any portion of that innate principle or essence remains in the habit. The circumstances above-mentioned, as well as others of a similar nature, seem to countenance, at least, such an opinion; and likewise to shew how tenaciously certain habits retain the relics of this essence, or with what difficulty it is entirely eradicated, the latter portions being thrown off, when a sufficient excitement has occurred to effect this, without any sensible excitement in the habit. The above facts likewise serve to confirm, if confirmation were wanting, that vaccination has a decided effect in ameliorating, to a great degree, or in disarming, small-pox of its natural virulence, should it happen afterwards, and of course removing any rational objection against the practice of vaccination.

My opinion, therefore, is, that vaccination, whether it be a modification of small-pox, or an affection *sui generis*, acts only in a partial or imperfect manner, in persons of a certain idiosyncrasy of habit, in securing them from future small-pox; and that such persons, if exposed to the small-pox infection some distant time afterwards, especially if that be of a virulent sort, and more particularly if this happen in hot weather, and in close contact, as in sleeping together, small-pox, under these circumstances, may occur. These circumstances I consider to have concurred, in a particular manner, in the family before-mentioned,—trusting, however, as this is the only instance I have met with myself, it will ever be an exceedingly rare occurrence, and consequently, considering the evident amelioration of the small-pox in consequence of prior vaccination, that such rare instances will never operate so far as to discourage vaccination, but rather to promote the practice of it.

The circumstance respecting vaccination, as a preventive of variolous infection, depending upon so many contingencies, entirely precludes any calculation, numerically, as to the extent of its preventive efficacy; but, probably, if variolous inoculation, the chief source of the propagation of natural small-pox, were entirely abandoned, and the practice of vaccination universal, small-pox, if not completely exterminated, would be an exceedingly rare occurrence.

What has happened at Crediton and Oxford, and probably bably in other places, which may not have come to my knowledge, may, and doubtless will, happen every where, should the same cause occur: hence no person, under such circumstances, need be surprised at it, but rest satisfied that vaccination still has the advantage of ameliorating the small-pox in those comparatively few in which it might fail as an absolute preventive; and, moreover, serve as a caution for persons not to expose themselves wantonly to the effect of a a more severe infection, such persons being certainly less secure than those who have passed through the variolous disease by inoculation. And practitioners must not, as heretofore, assure their patients, who have passed through vaccination in the most satisfactory manner, that they are absolutely incapable, at any future time, of taking small-pox; and must, in some degree, qualify that expression, according to the present established state of facts, as mentioned.

In short, cow-pox, I think, may be considered as a kind of Hybride species of small-pox, which certainly does not afford that degree of security from future small-pox which the legitimate disease itself does, and whoever imagines so, certainly over-rates its efficacy. Vaccination, which is probably a somewhat milder state of the same affection, and, perhaps, not equally efficacious with the original source of cow-pox, has, however, unquestionably, advantages which, in an eminent degree, recommend it to universal practice.

The practice of vaccination is, in reality, as simple, or easy, as that of inoculation; and, provided the operator apply the vaccine fluid in the state recommended above, in an effective manner, so as to produce an effect similar to what is before described, and caution his patient, or friends, respecting the *possibility* of taking future small-pox, the prudent means of avoiding it, and likewise the powerful effect of vaccination in ameliorating future small-pox, should it happen to follow,—he has done his duty; and, whatever may happen, no blame can justly attach to him.*

Anomalous eruptions, not referable to genuine small-pox, or to any other species of eruptive complaints, nor to any humour, as some persons suppose, imbibed by vaccination,

* Small-pox from inoculation may be considered as legitimate or genuine small-pox, but ordinarily in a milder state, owing to the mode by which it is communicated, and the preparatory means used by medicine and regimen; but there must ever remain, since the introduction of vaccination, an insuperable objection to inoculation, from which the former is free, viz. the *propagation of small-pox*, which is an evil every where to be dreaded, and as far as possible to be averted.

occasionally, occasionally, though rarely, occur, as I have experienced myself; but which are assignable to the imperfect nature of the affection, as mentioned above, and probably are the relics, somewhat changed, of legitimate small-pox; and, being neither dangerous nor permanent, and occurring but once, may be disregarded in practice.

Respecting the complete or perfect effect of vaccination, as far as it is capable of perfection, I shall present the following precautions. First, when the vesicle or pustule proceeds progressively on, and is accompanied by an efflorescent inflammation, extending to the size of a half-crown piece, or thereabout, about the tenth day, such effect may be considered as perfect, and it will scarcely happen but that some degree of indisposition, by attention, will be noticed about this time, or before. Secondly, if the vesicle or pustule go through its progress, as above-mentioned, but the efflorescent inflammation be entirely wanting, and no apparent indisposition, which will ordinarily be the case, such pustule may be considered as local only; and, though capable of communicating a perfect affection to another, is not to be considered as a security to the subject itself. Thirdly, if the part vaccinated becomes prematurely inflamed, viz. by the third or fourth day, and then quickly recedes and vanishes, such an appearance is perfectly ineffective, and may be considered what I shall call *abortive*.

In persons of a languid, cold, or leucophlegmatic habit, the efflorescent inflammation is ordinarily less bright than in persons of an ordinary habit. In persons of an opposite habit, viz. of an inflammatory diathesis, even in children, the efflorescent inflammation is sometimes so intense and extensive, as to create alarm in those unaccustomed to see it. In such cases, it may be adviseable to apply a cooling cerate, or poultice, to the part. This state of the part is, for the most part, very transient, rarely continuing beyond one day or so, although left to itself. If the part occasion uneasiness or inconvenience, by sticking to the linen, of course the application of some simple dressing will be necessary.

It will be found, by referring to my former papers on this subject, that the present is perfectly consonant with them, excepting somewhat of an abatement in my confidence respecting the degree of security from future small-pox by vaccination, my sentiments still remaining the same with respect to the propriety of persisting in its practice.

*August 4th,* 1815.

